# IL-33 in Mental Disorders

**DOI:** 10.3390/medicina57040315

**Published:** 2021-03-26

**Authors:** Gianluca Pandolfo, Giovanni Genovese, Marco Casciaro, Maria Rosaria Anna Muscatello, Antonio Bruno, Giovanni Pioggia, Sebastiano Gangemi

**Affiliations:** 1 Department of Biomedical and Dental Sciences, Morphological and Functional Images, University of Messina, 98125 Messina, Italy; gianluca.pandolfo@unime.it (G.P.); giovannigeno@live.it (G.G.); maria.muscatello@unime.it (M.R.A.M.); antonio.bruno@unime.it (A.B.); 2 School and Operative Unit of Allergy and Clinical Immunology, Policlinico “G. Martino”, Department of Clinical and Experimental Medicine, University of Messina, 98125 Messina, Italy; gangemis@unime.it; 3 Institute for Biomedical Research and Innovation (IRIB), National Research Council of Italy (CNR), 98125 Messina, Italy; giovanni.pioggia@cnr.it

**Keywords:** interleukin-33, alarmin, IL-33, inflammation, mental disorders, psychiatric, depression, bipolar, autism, ASD

## Abstract

Mental disorders are common in the general population; every year about 25% of the total European population is affected by a mental condition. The prevalence of psychiatric disorders might be underestimated. Emerging evidence highlights the role of immune response as a key factor in MDs. Immunological biomarkers seem to be related to illness progression and to treatment effectiveness; several studies suggest strong associations among IL-6, TNFa, S100b, IL 1b, and PCR with affective or schizophrenic disorders. The purpose of this review is to examine and to understand the possible link between mental disorders and interleukin 33 to clarify the role of this axis in the immune system. We found 13 research papers that evaluated interleukin 33 or interleukin 31 levels in subjects affected by mental disorders. Eight studies investigated cytokines in affective disorders. Three studies measured levels of IL-33 in schizophrenia and two studies focused on patients affected by autism spectrum disorders. Alterations in brain structure and neurodevelopmental outcome are affected by multiple levels of organization. Disorders of the autoimmune response, and of the IL-33/31 axis, may therefore be one of the factors involved in this process. These results support the evidence that alarmins, particularly the IL-33/31 axis, need more consideration among researchers and practitioners.

## 1. Introduction

Mental disorders (MDs) are common in the general population; every year about 25% of the total European population is affected by a mental condition (World Health Organization). According to other authors, the prevalence of psychiatric disorders might be underestimated [1]. MDs are a heterogeneous class of conditions, characterized by combination of symptoms that affect relationships, behavior, thoughts, perceptions, or emotion regulation [2]. The relationship between mental and physical aspects of health is complex, involving multiple levels of organization; mental health conditions are associated with disability and with an increased risk of a subsequent medical disease [3]. The biopsychosocial model is currently the main adopted perspective for MDs [4]. This model introduces the hypothesis that biological, psychological, and social determinants co-occur to define mental states. Most of the research on biological influences on MDs focus on genetic vulnerability and neurobiology (neurotransmitters, neuronal oscillation, and cortical networks); furthermore, the advances in neuroimaging research and molecular biology to characterize mental disorders has led researchers to a great effort to identify biomarkers in psychiatric conditions in order to clarify etiopathogenetic, diagnostic, and prognostic factors of the disease [5].

Emerging evidence highlights the role of immune response as a key factor in MDs. Immunological biomarkers seem to be related to illness progression and to treatment effectiveness; several studies suggest strong associations among IL-6, TNFa, S100b, IL 1b, and PCR with affective or schizophrenic disorders [6,7,8,9,10,11]. Yuan et al. (2019) evaluated the meta-analysis considering inflammatory markers in mental disorders. They concluded that there is a concrete possibility of differentiating the diseases by assessing the levels of the molecular mediators of inflammation [12]. Their conclusions were also supported by other studies which demonstrated how anti-inflammatory drugs were effective in ameliorating schizophrenia [13,14], on some others that reported how inflammatory mediators were suitable as markers in Major Depressive Disorder (MDD) [15]. Sterile inflammation induced by acute or chronic stress has been indicated as a possible etiopathogenetic mechanism for affective disorders, related to damage-associated molecular patterns (DAMP) signaling [16]. The receptor for advanced glycation end products (RAGE) has also been involved in stress-induced depression [17].

A recent theory highlights the role of the IL-33/IL-31 axis in different autoimmune disorders; immune system impairment as a comorbidity in psychiatric disorders was reported by several authors [18]. Alarmins are a multifunctional heterogeneous group of proteins, structurally belonging to specific cells or incorporated by them. They are released into the surrounding tissues as a consequence of tissue damage or inflammation. Their function is two-fold, as they could both activate the innate immunity and stimulate the adaptive immune response by recruiting and activating antigen-presenting cells (APC). Their apparently fundamental function is to ameliorate the immune system response, but on the other hand, the homeostasis could be compromised as they could easily lead to a large inflammatory response. They could generate a sort of vicious circle; in fact, alarmins are meant to be retained in the cytoplasm or to be secreted in certain dose and specific moments. A massive release of substances such as HMGB-1, S100, heat shock proteins, and others, after cells damage amplify massively the immune response, causing further damage, resulting in a proinflammatory loop. The latter effect could constitute the base for many diseases related to chronic inflammation such as COPD, asthma, heath failure, skin diseases, and neurologic disorders [19,20,21,22,23].

MD and autoimmune disease are frequently comorbid with each other and may share pathophysiological underpinnings. The immune response might be a shared etiopathogenetic mechanism, linking MDs with another medical condition. According to prior evidence, alarmins are interesting both for understanding inflammatory processes and for diagnostic purposes as biomarkers. Moreover, recent studies, separately, showed that alarmins like IL-33, HMGB1, HSP, and S100 could play a key role in the pathogenesis of mental disorders. For this reason, the purpose of this point of view is to examine and to understand the possible link between mental disorders and interleukin 33 to clarify the role of this axis in the immune system. A list of studies about IL-33 in psychiatric diseases is retrieved in Table 1.

## 2. Methods

This review was conducted by using Pubmed and Google Scholar databases. We entered the following key terms: alarmins, interleukin-33, IL-33, interleukin-31, IL-31, danger signals, AND mental disorders, psychiatric disorders, bipolar disorder, major depression, ASD, schizophrenia, autism. Only research articles written in English were included.

## 3. Results

According to literature, we found 13 research papers that evaluated interleukin 33 or interleukin 31 levels in subjects affected by mental disorders [24,25,26,27,28,30,31,34]. Eight studies investigated cytokines in affective disorders. Three studies measured levels of IL-33 in schizophrenia [29,35,36] and two studies focused on patients affected by autism spectrum disorders (ASD) [32,33].

### 3.1. Affective Disorders

Four studies investigated major depressive disorder (MDD) and other conditions, two studies investigated bipolar disorder (BD), one study MDD and BD, and one study depression and anxiety symptoms in patients affected by AA.

#### 3.1.1. Depression

Miller et al. examined the association between perinatal depression and cytokines [27]. They evaluated plasma and cerebrospinal fluid levels of IL-33 in a cohort of 76 pregnant woman with at least moderate depressive symptoms and not pharmacologically treated compared with euthymic women. The study sample consisted of 15 women affected by a major depressive episode (MDE), 11 women met the criteria for anxiety disorder, and the remainder of the women had subclinical symptoms. In term-pregnant women, higher levels of IL-33 in cerebrospinal fluid, but not in plasma, were significantly associated with an MDE after controlling for potential confounders. Ogłodek et al. investigated inflammatory biomarkers in depressed patients with and without post-traumatic stress disorder (PTSD) [28]. Four hundred and sixty people were recruited in the study, and subsequently divided into 7 groups each of 60 patients (30 males and 30 females) to assess mild depression (MiD), moderate depression (MoD), severe depression (SeD), MiD and PTSD, MoD and PTSD, SeD and PTSD, and PTSD alone. A MDE with or without PTSD was associated with high serum levels of IL-33. In PTSD comorbid with depression, IL-33 levels were higher than in depression alone. IL-33 levels were reported to increase with the severity of depression symptoms, as assessed with the Hamilton Depression Scale (HAM-D). No statistically significant differences were reported between males and females in any groups, even though females tended to have higher serum levels than males. Brunoni et al. in 2018 investigated response to treatment and IL-33 serum level in a placebo-controlled trial comparing transcranial direct current stimulation (tDCS) versus escitalopram efficacy in MDD, in 236 patients with low suicidal risk, at three timepoints during a 10-week treatment course [30]. According to their data, after 10 weeks IL-33 levels increased. Plasma levels of IL-33 were not associated with the outcome. Moreover, they searched for association between IL-33 plasma levels and age, not finding statistically significant results (age of participants from 18 to 75 years). Kudinova et al. (2016) studied the role of IL-33 as a potential risk factor for depression [31]. Two-single nucleotide polymorphisms (SNPs) haplotype in the IL-33 gene, specifically rs11792633 and rs7044343, moderated the link between women’s history of childhood abuse and their history of recurrent MDD (rMDD), such that the link between childhood abuse and rMDD was stronger among women with fewer copies of the protective IL-33 CT haplotype. Patients with a history of rMDD had higher peripheral levels of IL-33 and IL-1β compared to women with a single MDD episode or no history of MDD. Finally, in rats, an acute stressor increased IL-33 expression in the paraventricular nucleus of the hypothalamus and, to a lesser extent, the prefrontal cortex, key brain regions underlying stress response and emotion regulation. Bain et al. investigated the immune profiles of 39 patients affected by alopecia areata (AA) comorbid with anxiety and/or depression symptoms, compared to 23 patients affected by psoriatic arthritis and 26 healthy controls [26]. IL-33 and IL-31 were significantly higher in patients with AA than in HCs; there were no significative relationship between depression or anxiety with IL-31 or IL-33.

#### 3.1.2. Bipolar Disorder

Bavaresco et al. investigated inflammatory biomarkers in 36 adults patients affected by BD, at any stage of disease (manic, depressive, euthymic, or mixed episodes) compared to 46 healthy controls (HC), and their relation to biological rhythms [24]. They found no statistically significant difference between BD group and HC in blood serum levels of IL-33. Barbosa et al. in 2014 measured plasma levels of IL-33 and soluble ST2 (sST2), noticing increased circulating levels of the alarmin IL-33 in 46 patients with a bipolar disorder (23 in euthymia and 23 in mania) [34].

#### 3.1.3. Bipolar Disorders and Major Depressive Disorder

Brunoni et al. (2020) investigated possible differences in immune profiles between patients affected by BD and patients affected by MDD [25]. They tested 245 patients suffering from MDD and 59 patients suffering from BD, recruited during an acute depressive episode of moderate severity. Plasma levels of IL-33 and its soluble receptor, sST2, were significantly higher in BD than MDD; sST2 works sequestering extracellular IL-33. This soluble receptor was associated to pathological states and dosing it could be as important as dosing IL-33 for disease staging and progression.

### 3.2. Schizophrenia

Three studies investigated the level of IL-33 in schizophrenia. Subbanna et al. investigated schizophrenia biomarkers in a sample of 27 drug-naive schizophrenia patients, and their variation before and after antipsychotic medications. They found no statistically significant differences in IL-33 plasma levels after 3 months of treatment [36]. Borovcanin et al. investigated serum level of IL-33 and sST2 in different stages of schizophrenia [29]; 167 participants were evaluated: 77 drug naïve patients with first episode psychosis (FEP), 45 relapsed schizophrenia patients with acute symptoms, 27 remitted schizophrenia patients, and 18 HC. IL-33 serum levels did not differ between FEP and relapsed SC group, and between remitted SC and HC subjects. FEP and relapsed SC patients had significantly higher IL-33 sera levels compared to remitted SC patients and HC. The authors observed a descending gradient of serum levels of sST2 with higher levels in patients with FEP, followed by relapsed SC, and remitted SC, with lower levels found in control subjects. Differences in serum levels of sST2 between groups were statistically significant except between patients with remitted SC in remission and HC group. In relapsed SC group, sST2 serum levels negatively correlated with PANSS negative subscale scores, whereas in remitted SC patients IL-33 serum levels resulted significantly correlated with PANSS positive symptoms, especially hostility (item P7). De Campos-Carli et al. (2017) analyzed IL-33 and ST2 levels of 40 schizophrenic subjects compared with healthy people, finding no differences between groups [35]; nevertheless, increased levels of both IL-33 and sST2 were associated with better performances in in multiple cognitive domains, such as verbal memory, semantic verbal fluency attention, and processing speed.

### 3.3. Autism Spectrum Disorders

Two studies investigated IL-33 in ASD. Saresella et al. observed that an increased production of IL-1β and IL-18 was associated with a consistent reduction of IL-33 in ASD [32]. Barbosa et al. evaluated the plasma levels of IL-33, sST2, and IL-1β in ASD patients and healthy controls; ASD alarmin levels did not differ between the two groups [33].

## 4. Discussion

### IL-33, Mental Health and Immune System

The influence of the immune system on mental diseases onset and progression remains controversial. During the last decade, it emerged the importance of two phenomena in the development of psychiatric disorder: oxidative stress and inflammation, two processes that are intimately linked. In several other papers from our group, we described the chronic inflammatory loop deriving from oxidative stress [22,37]. In a previous manuscript, our group showed the involvement of alarmins and, in particular, interleukin 33, in the development of ASD, sustaining systemic and consequently brain inflammation. Our hypothesis consisted of the individualities of innate type 2 lymphoid cells (IL-C2s), which increased after IL-33 stimulation, as the key player in this cascade. These cells, in fact, cause the release of proinflammatory cytokines, starting a type-2 inflammation. This mechanism was also corroborated by results correlating high brain IL-33 levels to behavioral disturbances [38]. In the present review, we collected interleukin 33 data in main psychiatric disorders such as MDD, BD, schizophrenia, ASD, anxiety, and PTSD. IL-33 resulted constantly augmented only in MDD and reduced in ASD. In BD and schizophrenia, the results were conflicting, suggesting once more the need for more systematic and larger studies, considering the diverse phases of the disease. Data on MDD seem to be clear, but a recent research conducted by our group revealed that postmenopausal women had lower levels of the abovementioned alarmin [39]. Thus, a question arises when we consider that often peri-postmenopausal women have a higher incidence of depression symptoms [40]. Probably the answer may consist in the fact that the women considered in our paper were affected by osteoporosis, but not by depression and that parathormone and vitamin D together with bone remodeling may have influenced the levels of the interleukin. Future research could be focused on the analysis of IL-33 and other inflammatory cytokines in relation to depression symptoms in the postmenopausal period, with particular attention given to the nutritional state of the women considered. It could be interesting testing whether or not they lack vitamins and antioxidants that could influence the inflammatory status and consequently mental health.

The analysis of IL-33 role in animal behavior revealed that mice lacking of the interleukin undergo to a reduction of anxiety, but they also develop an impaired social recognition. The authors speculated a key role of IL-33 in the neurodevelopment due to its potential action on both axons and glial cells and its action in the postnatal brain dynamics [41]. As noted above, sST2 is the soluble receptor of IL-33, and it is a key molecule for the immune system too. One researcher revealed that sST2 overexpression in mice resulted in decreased social interaction [42].

**Figure 1:**
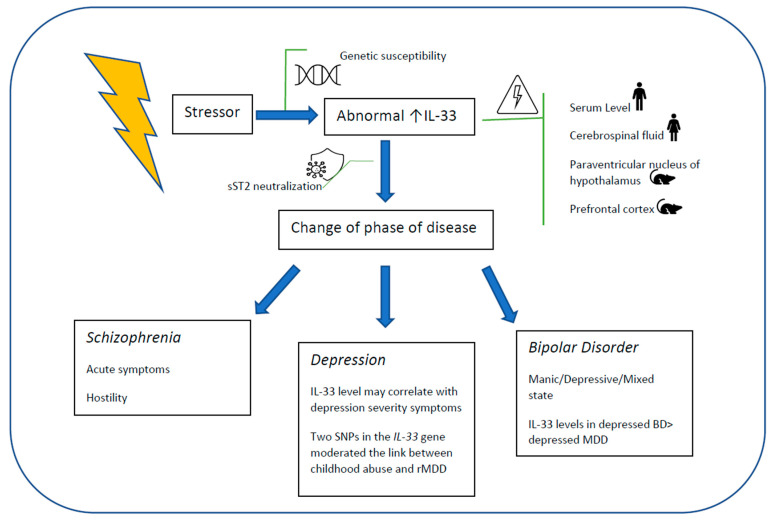
IL-33 levels correlated to disease phases after the intervention of a stressing agent.

Taken together, the results reviewed support the evidence that elevated levels of IL-33 are associated with acute symptoms in affective disorders and schizophrenia, and with recurrence in depression (Figure 1).

According to the literature, and from a speculative point of view, we may hypothesize that IL-33/IL-31 axis is involved in processes related with relapses and recurrence of mental disorders; within this framework, future developments in telemedicine may consider IL-33 measurements as a potential biomarker for monitoring the course of psychiatric disorders. On the other hand, to date, IL-33 does not match as a biomarker for predicting response to treatment, probably due to some bias on the selected cohort of patients or to the timing of the sample collection during the few studies previously conducted.

## 5. Conclusions

From the etiopathogenetic point of view, psychiatric disorders are currently seen as complex disorders in which one or various alterations, acting sooner or later in time, can lead to alterations of development trajectories and mental functioning with different phenomenological expression. Alterations in brain structure and neurodevelopmental outcome are affected by multiple levels of organization. Disorders of the autoimmune response, and of the IL-33/31 axis may therefore be one of the factors involved in this process. As a whole, these results support the evidence that alarmins, particularly the IL-33/31 axis, need more consideration among researchers and practitioners. Most of the studies evaluated IL-33 or IL-31 as part of a broader assessment of various inflammatory biomarker. This approach leads to a lack of studies focused on the IL-33/31 axis. The path to further studies to better define the role of alarmins in mental disorders needs to be built around specific psychiatric variables as age, phase of disease, age from onset, number of acute episodes, comorbidity, drugs used, and immunological variables such as sST2 and IL-31. IL-33 serum levels might be a modifiable risk factor for increased disability, chronicization, and medication overuse.

If confirmed by more systematic studies in larger cohort of patients, the analysis of IL-33 and IL-31, and their reciprocal ratio together with the levels of soluble receptors, could be an effective method for disease staging, whereas the role on treatment efficacy evaluation needs further data. On the other hand, animal studies should also be considered as equally important, as they are fundamental for understanding the etiopathogenesis of the disease and how to reach an effective inflammatory balance. Furthermore, acting both on inflammation by using anti-inflammatory medications and by evaluating novel monoclonal antibodies could be the key for future approaches to mental disorders. Understanding the exact inflammatory status in psychiatric patients could, in the medical approach of the future, help the psychiatrist in the therapeutic decisional process. Treatment approach should consist of psychiatric drugs, but it also should aim to reduce chronic inflammation in order to ameliorate patient status.

## Figures and Tables

**Table 1:** The table retrieves the manuscripts evaluating IL-33 in mental disorders.

Reference	Objective	Mental Disorder	Subjects	Findings
[24]	Biological rhythms in bipolar disorder (BD) and inflammatory biomarkers	BD	36 BD and 46 healthy controls	No statistically significant differences in IL-33 (interleukin 33) levels between the BD group and the control group (*p* value = 0.959)
[25]	Differences in immune profiles between BD and major depressive disorder (MDD) patients	MDD, BD	245 MDD and 59 BD patients in an acute depressive episode of moderate severity	IL-33 was significantly higher in BD than MDD (*p* < 0.001)
[11]	Biomarkers and antipsychotic medications	Schizophrenia (SCZ)	27 drug-naive schizophrenia patients	no statistically significant difference in IL-33 plasma levels before and after treatment.
[26]	To investigate the association between cytokines and depression in patients affected by alopecia areata (AA)	Anxiety and Depression symptoms	39 patients with AA, 23 Psoriatic arthritis, 26 healthy controls	IL-33 and IL-31 were significantly higher in patients with AA than HCs, no significative correlations with psychiatric symptoms.
[27]	Perinatal depression and cytokine levels in plasma and cerebrospinal fluid	Perinatal Depression	76 patients with depressive symptoms without AD medications	In term pregnant woman IL-33 was significantly associated with an MD episode.
[28]	Biomarkers in depressed patient with and without post-traumatic stress disorder (PTSD)	Depression comorbid with PTSD	Each study group comprised 60 patients: mild depression (MiD), moderate depression (MoD), severe depression (SeD), MiD and PTSD (MiD + PTSD), MoD and PTSD (MoD + PTSD), SeD and PTSD (SeD + PTSD), PTSD, and 40 HC	Depression alone or comorbid with PTSD was associated with high levels of IL-33, with higher levels when in presence of comorbid PTSD. IL-33 levels were reported to increase as depression became more severe both in males and females. Although not significant, there was a trend in females towards higher concentration levels of this parameter than males.
[29]	IL-33 and soluble ST2 (sST2) in different stages of schizophrenia	SCZ	77 drug naïve patients with first episode psychosis (FEP) 45 schizophrenia in relapse (SC in relapse) 27 schizophrenia in remission (SC in remission) 18 healthy controls (HC)	- IL-33 and sST2 serum levels were higher in schizophrenia exacerbation; - sST2 serum levels negatively correlated with negative symptoms in acute psychosis; - in SC in remission serum IL-33 and sST2 correlated with positive symptoms, especially hostility.
[30]	To investigate plasma biomarkers in a placebo-controlled trial comparing tDCS and escitalopram efficacy in major depression	MMD	236 patients	No association between IL-33 levels and depression improvement, also controlling for age.
[31]	Data from three complimentary studies that support the role of, interleukin-33 in depression risk	MMD (single episode vs. recurrent)	Study 1: recurrent MDD = 76 single episode of MDD = 40 no MDD *n* = 125; Study 2: recurrent MDD = 10 single episode of MDD = 10 no MDD = 20	two-SNP haplotypes in the IL-33 gene (rs11792633 and rs7044343) moderated the link between women’s history of childhood abuse and history of recurrent MDD. Patients with recurrent MDD had higher peripheral levels of IL-33 and IL-1β compared to women with a single MDD episode or no history of MDD. Acute stressor increased IL-33 expression in the paraventricular nucleus of the hypothalamus.
[32]	To investigate the inflammasomes activity in autism spectrum disorders (ASD)	ASD	25 ASD 23 unaffected siblings of the ASD patients 30 HC	Increased production of IL-1β and IL-18 that was associated with a consistent reduction of IL-33 in ASD
[33]	To evaluate plasma levels of IL-33 in ASD	ASD	30 patients with ASD 18 HC	Patients did not differ from controls in IL-33 levels
[34]	Plasma levels of IL-33 and ST2 in bipolar disorder	Bipolar disorder	46 BD patients	Increased plasma levels of IL-33 in bipolar disorder
[35]	Involvement of IL-33 in schizophrenia and its association with cognitive performance	Schizophrenia	40 patients 40 controls	Patients with schizophrenia and controls presented similar serum levels of IL-33 and sST2. Levels of both markers were positively correlated with better cognitive performance in patients with schizophrenia.
